# Open-Label Multicenter Registry on the Outcomes of In-Stent Restenosis Treated by Balloon Angioplasty with Optical Frequency Domain Imaging in the Superficial Femoral Artery (ISLAND-SFA Study)

**DOI:** 10.3400/avd.oa.20-00077

**Published:** 2020-09-25

**Authors:** Kenichi Yanaka, Akihide Konishi, Toshiro Shinke, Amane Kozuki, Hiroyuki Kawamori, Yoshiro Tsukiyama, Osamu Iida, Makoto Kadotani, Takashi Omori, Ken-ichi Hirata

**Affiliations:** 1 Division of Cardiovascular Medicine, Department of Internal Medicine, Kobe University Graduate School of Medicine; 2 Clinical & Translational Research Center, Kobe University Hospital; 3 Division of Cardiology, Department of Medicine, Showa University School of Medicine; 4 Division of Cardiology, Osaka Saiseikai Nakatsu Hospital; 5 Cardiovascular Center, Kansai Rosai Hospital; 6 Kakogawa Central City Hospital

**Keywords:** optical frequency domain imaging, balloon angioplasty, recurrent restenosis, in-stent restenosis, superficial femoral artery

## Abstract

**Objectives**: Balloon angioplasty for in-stent restenosis (ISR) in the superficial femoral artery (SFA) has a high recurrent restenosis rate; however, its mechanism has not been fully and precisely evaluated using high-resolution intravascular imaging. Thus, we aimed to evaluate the relationship between vascular features obtained by optical frequency domain imaging (OFDI) and recurrent restenosis at 6 months.

**Methods**: This was a prospective multicenter single-arm study. OFDI was performed before and after balloon angioplasty, and vascular features were assessed. A multi-layered ISR pattern detected by OFDI was defined as several signal-poor appearances with a high-signal band adjacent to the luminal surface. The primary outcome was defined as recurrent restenosis 6 months after balloon angioplasty.

**Results**: Given that this study was terminated early, only 18 patients completed the 6-month follow-up; of these, 8 developed restenosis. Recurrent restenosis at 6 months tended to be related to a multi-layered ISR pattern (odds ratio (OR), 6.67; 95% confidence interval (CI), 0.81–54.96; p=0.078) and the minimum lumen area (MLA) after balloon angioplasty (OR, 0.71; 95%CI, 0.48–1.04; p=0.077).

**Conclusion**: A multi-layered ISR pattern and MLA after balloon angioplasty detected by OFDI might be risk factors for recurrent ISR in the SFA.

## Introduction

Endovascular treatment (EVT) is the first-line approach for the revascularization of symptomatic femoropopliteal artery disease.^1,2)^ Multiple modalities exist, but balloon angioplasty, rather than additional stent implantation, is one of the main strategies of EVT for in-stent restenosis (ISR) in the superficial femoral artery (SFA).^3,4)^ Although balloon angioplasty without stenting for ISR lesion has been associated with recurrent restenosis rates of up to 70% at 12 months,^3,5)^ the mechanism of the high recurrent restenosis rates after balloon angioplasty has not been fully and precisely evaluated using high-resolution intravascular imaging.

Given that a number of different competing treatment modalities, including covered stents, drug-eluting stents, and drug-coated balloon, have been developed, and multiple trials were performed to evaluate them, typically comparing those with balloon angioplasty,^6)^ it would be important to approach this topic with the attitude of looking back to the past to inform the future.

Intravascular optical coherence tomography (OCT) has emerged as a high-resolution intracoronary imaging modality, providing microscopic images of intravascular features,^7)^ but it has a lower penetration depth compared with intravascular ultrasound. Recently, second-generation optical frequency domain imaging (OFDI) has been developed to provide greater penetration depth and higher pullback speed.^8,9)^

The aim of this study was to evaluate the relationship between the vascular features obtained by OFDI before and after balloon angioplasty in ISR in the SFA and recurrent restenosis 6 months after balloon angioplasty.

## Methods

### Study design

This prospective multicenter single-arm study was conducted at the following four institutes in Japan: Kobe University Hospital, Osaka Saiseikai Nakatsu Hospital, Kansai Rosai Hospital, and Hyogo Prefectural Awaji Medical Center. In this prospective interventional exploratory study, patient enrollment was planned from April 4, 2016 to September 30, 2018. The follow-up period was 6 months for each patient. The study protocol was approved by the ethical review board of each participating institution. The study was registered in the UMIN clinical trial registry (UMIN000021121) and approved by the institutional review boards at Kobe University Hospital (approval no. 270034), Osaka Saiseikai Nakatsu Hospital (approval no. H28-029), Kansai Rosai Hospital (approval no. 16C071g), and Hyogo Prefectural Awaji Medical Center (approval no. 29-13). Written informed consent to participate in this study was obtained from all patients.

### Participants

#### Inclusion criteria

Patients had to satisfy the following criteria to be included in the study: 1) patients with ISR in the SFA, 2) patients with peripheral arterial disease (Rutherford 1–5 categories), 3) patients meeting the endovascular therapy criteria, 4) patients who understood the study protocol and provided written informed consent, and 5) patients aged >20 years at the time of obtaining informed consent.

#### Exclusion criteria

The following were excluded: 1) patients with in-stent occlusion in the SFA, 2) patients with occlusion of all three arteries below the knee, 3) patients with uncontrollable heart failure, 4) patients with acute limb ischemia or acute thrombotic occlusion, 5) patients contraindicated for antiplatelet therapy, 6) pregnant or breast-feeding women, and 7) patients judged by a physician to be unfit to participate in this study.

### Interventions

When the procedure was optimally completed as assessed by angiography, OFDI was performed before, and after balloon angioplasty. The stent may be placed at the discretion of the surgeon in case the procedure was not optimally completed. The treatment was performed in accordance with actual clinical practice.

### OFDI examination

The OFDI examination was performed using TE-200 (LUNAWAVE®: imaging system, Terumo Corporation, Tokyo, Japan). Although OFDI is indicated for percutaneous coronary intervention, it is not indicated for EVT in Japan.

After a manual calibration, the OFDI catheter is advanced >5 mm distally to the target lesion over a 0.014-inch conventional angioplasty guidewire. Following the catheter placement, 50% of the contrast media is flushed through the guiding catheter using a 30 or 50 ml syringe with manual injection and manual compression of the femoral artery at the treatment side to remove blood flow. When a blood-free image is observed, the OFDI catheter is pulled back over a longitudinal distance of up to 150 mm at a rate of 40 mm/s using a stand-alone electronic control of the motor drive unit. The total volume of the contrast media for EVT and that of the media used to capture OFDI images were recorded.

### OFDI analysis

All images obtained from OFDI were analyzed by independent observers who were blinded to the clinical presentations and lesion characteristics. Cross-sectional OCT images were analyzed at 5 mm intervals.

**Figure 1:**
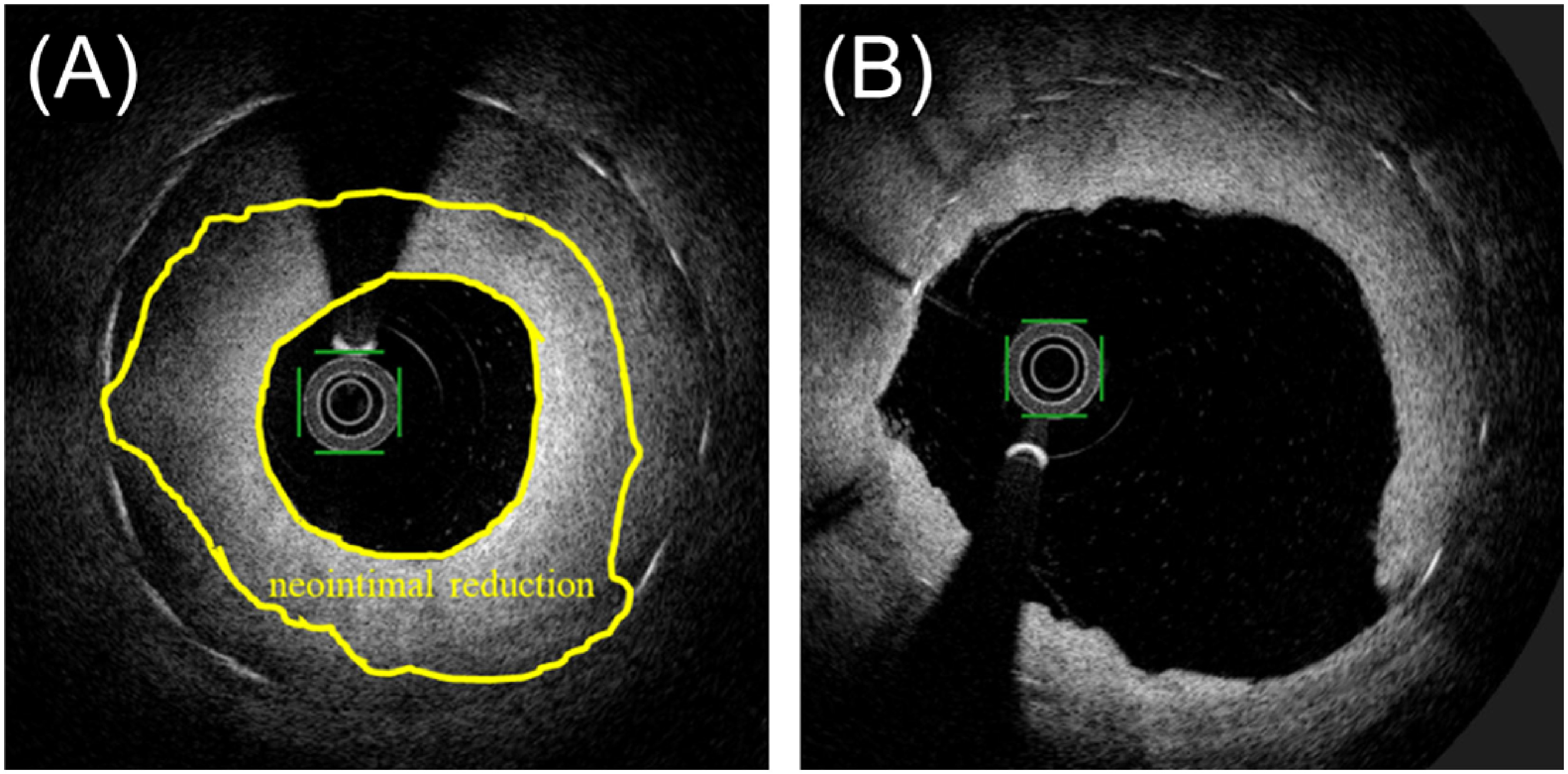
Fig. 1 A schematic illustration of the measurements of neointimal reduction. (**A**) pre-balloon dilatation, (**B**) post-balloon dilatation.

In the quantitative analysis, lesion length; maximum, average and minimum stent areas; maximum, average, and minimum lumen areas (MLAs); and acute gain were measured manually both before and after balloon angioplasty. The neointimal area was defined as the stent area minus the lumen area. Neointimal reduction was defined as the average neointimal area of pre-balloon angioplasty minus that of post-balloon angioplasty (**Fig. 1**). Struts were classified as covered if a tissue layer was visible over all reflecting surfaces. The frequency of the covered struts was calculated as the number of those struts divided by the total number of struts for each stent. Neointimal thickness was measured from the center reflection of the stent strut to the vessel lumen border (neointimal surface or strut surface if uncovered) for each stent strut. Maximum, average, and minimum dissection flap areas after balloon angioplasty were assessed.

**Figure 2:**
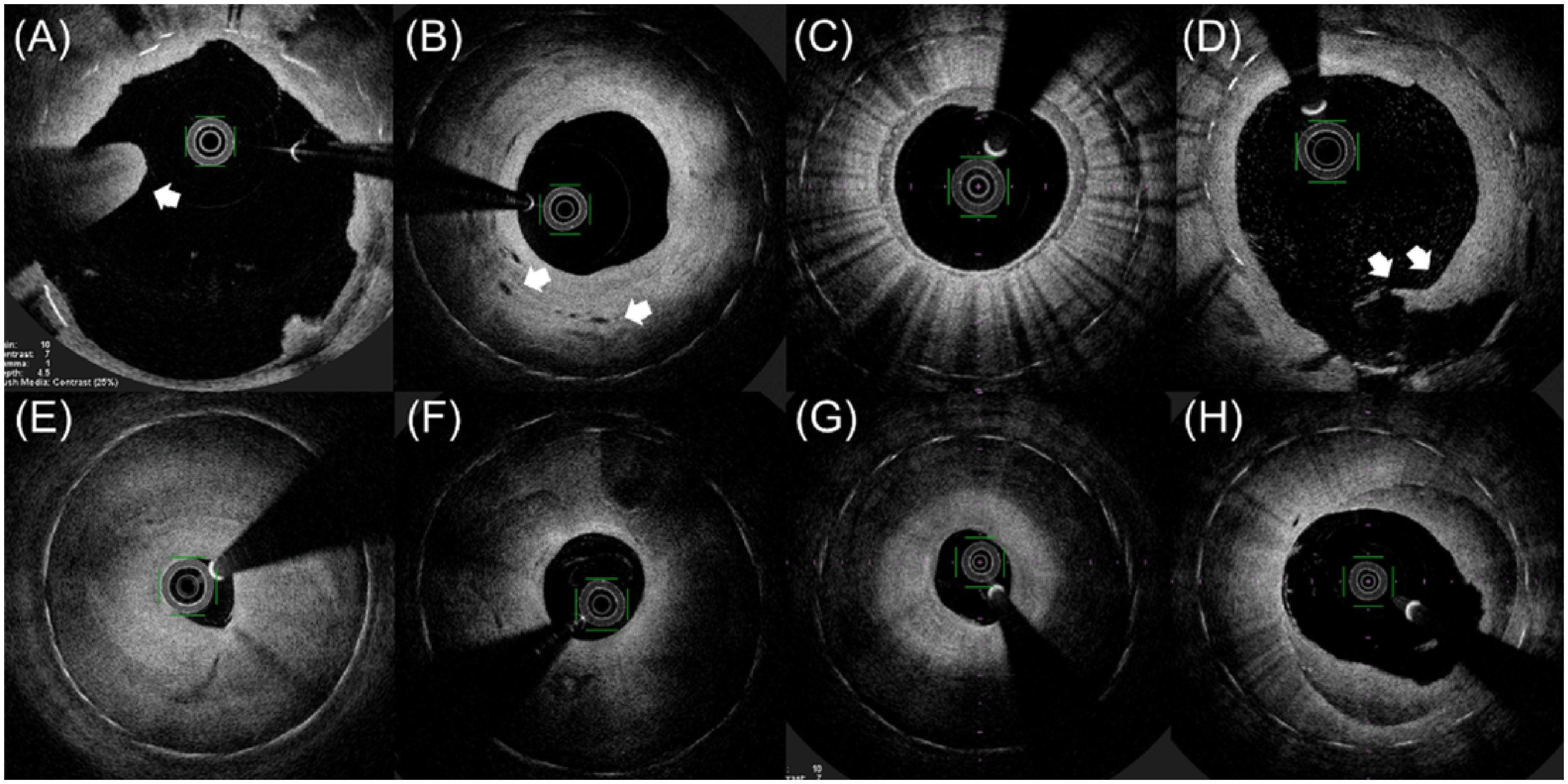
Fig. 2 Representative optical coherence tomography images. (**A**) thrombus; (**B**) vasa vasorum; (**C**) macrophage accumulation; (**D**) major dissection; (**E**) homogeneous ISR pattern; (**F**) heterogeneous ISR pattern; (**G**) layered ISR pattern; (**H**) multi-layered ISR pattern.

In a qualitative analysis, thrombus, vasa vasorum, and macrophage accumulation were evaluated. Thrombus was defined as a protruding irregular luminal mass of >250 µm in maximal height with dorsal shadowing that is not connected to the vessel wall^10)^ (**Fig. 2**). Vasa vasorum was defined as micro-vessels that can be clearly visualized within the plaque (**Fig. 2**). Macrophage accumulation was defined as a neointima containing a diffuse border and a poor signal region with invisible struts due to marked signal attenuation (**Fig. 2**). Neointimal thickness was classified into the following four patterns based on Gonzalo’s classification^11)^: homogeneous, layered, multi-layered,^12)^ and heterogeneous patterns (**Fig. 2**). The homogeneous pattern was characterized by a uniform signal-rich appearance, containing a mostly signal-rich structure. The layered pattern had one signal-poor appearance with a high-signal band adjacent to the luminal surface. The multi-layered pattern had several signal-poor appearances with a high-signal band adjacent to the luminal surface. The heterogeneous pattern mainly consisted of signal-poor appearances with islands of various signal regions.

ISR: in-stent restenosis

### Outcomes

The primary outcome of this study is the recurrent restenosis detected by duplex ultrasonography 6 months after balloon angioplasty. The definition of recurrent restenosis was based on the physician’s judgment on the referred peak systolic velocity ratio (PSVR), whereas in the protocol, it was described as the use of only PSVR. The secondary outcome was the recurrent restenosis detected by duplex ultrasonography at 12 months.

### Sample size

Based on a previous study,^3)^ the proportion of patients with recurrent restenosis 6 months after treatment was approximately 70%. The proportion of patients with recurrent restenosis at 6 months was 96% in a group dichotomized by an imaging parameter and 56% in another group, with an alpha error of 5% and power of 80%, and the ratio of the groups of 2 : 1; thus, the required sample size would be 50 patients. Considering the drop-out, we considered that we had to enroll 60 participants.

### Analysis population

This study’s analysis population consisted of all patients enrolled in this study.

### Statistical methods

The clinical characteristics of the participants were summarized as mean and standard deviation for quantitative variables and as frequency and proportion for qualitative variables.

As primary outcomes, recurrent restenosis 6 months after balloon angioplasty, summary statistics of the clinical characteristics, and OFDI imaging parameters according to the presence and absence of recurrent restenosis were assessed. The odds ratios (ORs) and 95% confidence intervals (CIs) were estimated using a simple logistic regression model. P-values for OR were estimated, without any adjustment for multiplicity. Cases with missing data were deleted from the dataset used in the analyses.

For OFDI parameters of the ISR pattern, we redefined the complete homogeneous ISR pattern as having only a homogeneous pattern, without a heterogeneous, layered, or multi-layered appearances. The relationship between recurrent restenosis at 6 months and neointimal reduction was examined in the subgroup of patients with a complete homogeneous ISR pattern.

All statistical analyses were conducted using SAS software (version 9.4, SAS Institute, Cary, NC, USA).

## Results

### Early termination of the study

The study was terminated early on March 30, 2019, because its principal investigator left the institute and the continuation of the study required reconsideration. In accordance with the protocol, the Safety Evaluation Committee was consulted regarding early termination and found that the decision was valid. The last patient was entered on May 11, 2018, and a total of 23 patients were enrolled in this study.

### Participant flow and baseline demographic and clinical characteristics

**Figure 3:**
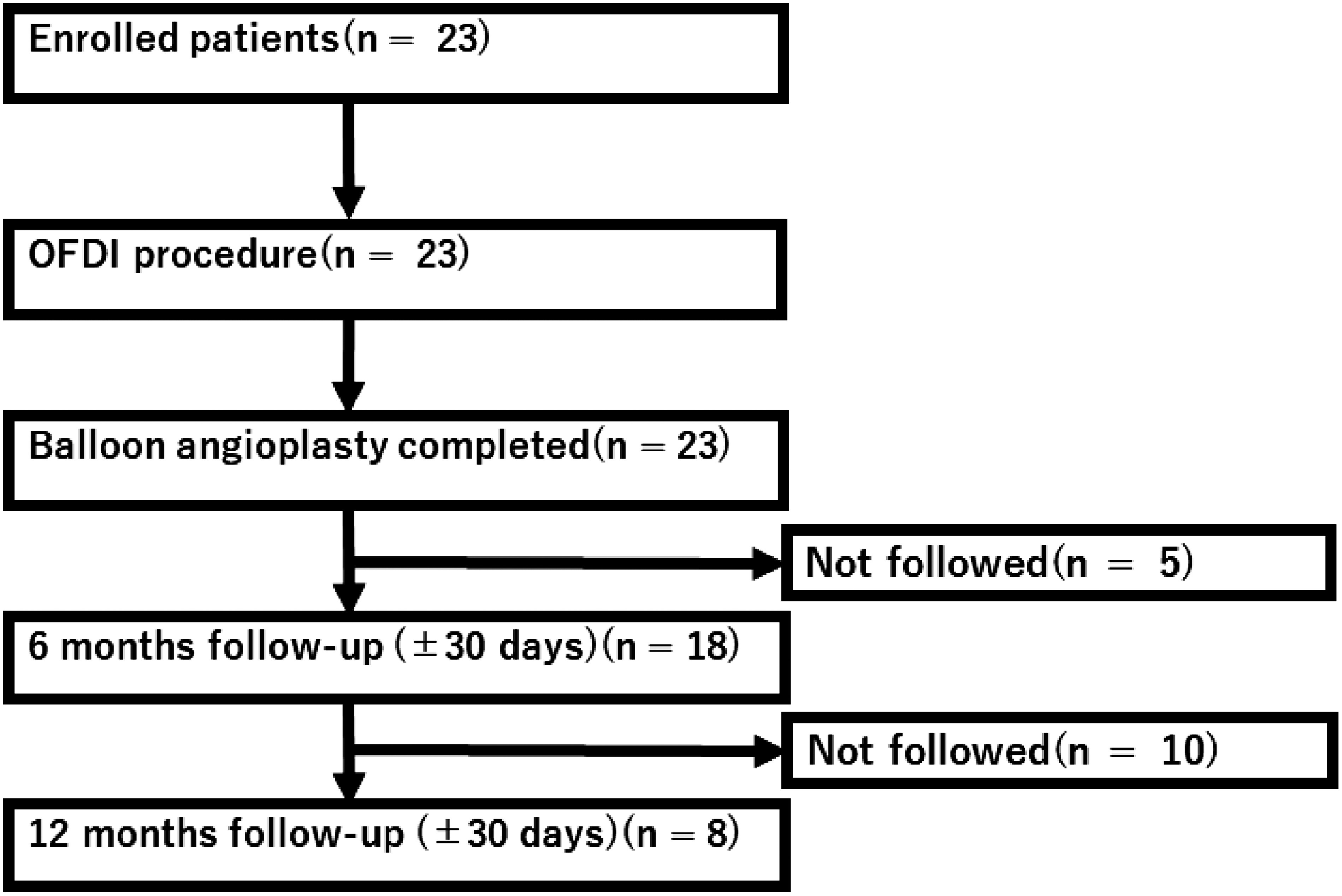
Fig. 3 Flowchart of patients included in the study.

**Figure 3** presents the participant flow. A total of 18 participants were followed up and assessed for recurrent restenosis 6 months after the balloon angioplasty. All patients had received antiplatelet drugs. Given that the number of participants followed up until 12 months was only 8, we will not report for the secondary outcome.

OFDI: optical frequency domain imaging

The clinical characteristics are summarized in **Table 1**.

**Table 1:** Table 1 Association between participant characteristics and recurrent stenosis 6 months after balloon angioplasty

	All^a^ (n=18)	Recurrent restenosis 6 months after balloon angioplasty				
		Presence^a^ (n=8)	Absence^a^ (n=10)	OR	95%CI	p-value^b^
					[Lower, Upper]	
Age (years)	74.8±8.06 (18)	75.6±9.2 (8)	74.1±7.5 (10)	1.03	[0.91, 1.16]	0.683
Female sex (%)	6 (33.3%)	4 (50.0%)	2 (20.0%)	4.00	[0.50, 31.98]	0.191
Height (cm)	158.9±10.7 (18)	155.8±12.1 (8)	161.3±9.4 (10)	0.95	[0.86, 1.05]	0.281
Weight (kg)	57.1±11.2 (18)	53.3±8.1 (8)	60.1±12.8 (10)	0.94	[0.85, 1.04]	0.210
BMI (kg/m^2^)	22.5±3.1 (18)	22.1±3.4 (8)	22.9±3.0 (10)	0.92	[0.67, 1.25]	0.580
Diabetes (%)	11 (61.1%)	4 (50.0%)	7 (70.0%)	0.43	[0.06, 2.97]	0.391
Hypertension (%)	14 (77.8%)	7 (87.5%)	7 (70.0%)	3.00	[0.25, 36.33]	0.388
Dyslipidemia (%)	14 (77.8%)	7 (87.5%)	7 (70.0%)	3.00	[0.25, 36.33]	0.388
Smoking (%)	13 (72.2%)	5 (62.5%)	8 (80.0%)	1.07	[0.77, 1.47]	0.699
Dialysis (%)	2 (11.1%)	0 (0%)	2 (20.0%)	—	[—, —]	—
Coronary stenosis (%)	17 (94.4%)	7 (87.5%)	10 (100.0%)	—	[—, —]	—
History of stroke (%)	0 (0.0%)	0 (0.0%)	0 (0.0%)	—	[—, —]	—
History of heart failure (%)	3 (16.7%)	0 (0.0%)	3 (30.0%)	—	[—, —]	—
RBC (×10^4^/mL)	416.4±54.6 (18)	398.8±55.8 (8)	430.6±52.0 (10)	0.99	[0.97, 1.01]	0.220
WBC (/mL)	6,516.7±2,041.7 (18)	6,825.0±2,241.0 (8)	6,270.0±1,953.3 (10)	1.00	[1.00, 1.00]	0.559
PLT (×10^4^/mL)	22.4±6.1 (18)	22.6±6.7 (8)	22.2±6.0 (10)	1.01	[0.87, 1.18]	0.892
Hb (g/dL)	12.3±1.4 (18)	12.1±1.6 (8)	12.5±1.3 (10)	0.82	[0.41, 1.67]	0.591
Ht (%)	38.1±4.3 (18)	37.1±4.7 (8)	38.9±4.0 (10)	0.90	[0.71, 1.13]	0.367
Cr (mg/dL)	1.5±1.7 (18)	0.9±0.3 (8)	1.9±2.2 (10)	0.46	[0.09, 2.51]	0.372
TG (mg/dL)	136.1±76.8 (16)	143.0±82.7 (7)	130.8±76.5 (9)	1.00	[0.99, 1.02]	0.745
Total chol (mg/dL)	179.0±45.7 (16)	191.6±64.0 (7)	169.2±24.3 (9)	1.01	[0.99, 1.04]	0.348
HDL chol (mg/dL)	54.0±36.2 (16)	56.9±21.5 (7)	51.8±14.8 (9)	1.02	[0.96, 1.08]	0.558
LDL chol (mg/dL)	97.3±36.2 (16)	105.4±52.0 (7)	91.0±17.9 (9)	1.01	[0.98, 1.05]	0.438

The second column presents the enrolled patients’ characteristics. The characteristics according to restenosis 6 months after balloon angioplasty are shown in the third and fourth columns. The odds ratio as a measurement of the relationship between restenosis 6 months after balloon angioplasty and each patient characteristic, along with its 95% confidence interval and p value, are displayed in the fifth, sixth, and seventh columns. ^a^ Mean±standard deviation (n) for continuous variables; frequency count (%) for the categorical variable. ^b^ p-values were calculated using a simple logistic regression. BMI: body mass index; RBC: red blood cells; WBC: white blood cells; PLT: platelets; Hb: hemoglobin; Ht: hematocrit; Cr: creatinine; TG: triglyceride; Total chol: total cholesterol; HDL chol: high-density lipoprotein cholesterol; LDL chol: low-density lipoprotein cholesterol; OR: odds ratio; 95%CI: 95% confidence interval; Lower: lower limit; Upper: upper limit

### OFDI imaging parameters

**Table 2** lists the summary statistics of the investigated OFDI imaging parameters.

**Table 2:** Table 2 Association between OFDI parameters and recurrent restenosis 6 months after balloon angioplasty

	All^a^ (n=18)	Recurrent restenosis at 6 months after balloon angioplasty				
		Presence^a^ (n=8)	Absence^a^ (n=10)	OR	95%CI	p-value^b^
					[Lower, Upper]	
(Before balloon angioplasty)						
Lesion length, mm	110.4±73.9 (18)	142.5±96.1 (8)	84.7±38.5 (10)	1.01	[1.00, 1.03]	0.128
Lumen area, mm^2^						
Maximum	20.4±8.3 (18)	20.6±8.3 (8)	20.2±8.7 (10)	1.01	[0.90, 1.13]	0.917
Minimum	4.3±3.5 (18)	3.0±1.6 (8)	5.4±4.3 (10)	0.76	[0.52, 1.13]	0.171
Average	11.6±5.0 (18)	10.9±4.3 (8)	12.2±5.6 (10)	0.94	[0.77, 1.15]	0.558
Stent area, mm^2^						
Maximum	36.9±9.7 (18)	35.7±10.6 (8)	38.0±9.3 (10)	0.97	[0.88, 1.08]	0.605
Minimum	24.6±6.2 (18)	25.2±6.4 (8)	24.0±6.2 (10)	1.03	[0.88, 1.21]	0.673
Average	30.5±6.6 (18)	28.6±5.9 (8)	32.2±7.1 (10)	0.91	[0.78, 1.07]	0.252
Average neointimal thickness, mm	1.2±0.3 (18)	1.2±0.2 (8)	1.3±0.41 (10)	0.46	[0.03, 8.51]	0.601
Average neointimal area, mm^2^	18.9±5.1 (18)	17.7±3.6 (8)	19.9±6.0 (10)	0.91	[0.74, 1.11]	0.343
Neointimal reduction, mm^2^	5.7±3.2 (18)	4.2±1.8 (8)	6.9±3.6 (10)	0.71	[0.49, 1.04]	0.082
Percent of covered strut, %	99.9±0.3 (18)	100.0±0.1 (8)	99.8±0.4 (10)	17.8	[0.03, >0.99]	0.374
Acute gain, mm	8.9±4.7 (18)	7.5±3.4 (8)	10.1±5.4 (10)	0.87	[0.68, 1.10]	0.247
Layered ISR pattern, %						
Presence	7 (38.9%)	4 (50.0%)	3 (30.0%)	2.33	[0.34, 16.18]	0.391
Absence	11 (61.1%)	4 (50.0%)	7 (70.0%)			
Multi-layered ISR pattern, %						
Presence	7 (38.9%)	5 (62.5%)	2 (20.0%)	6.67	[0.81, 54.96]	0.078
Absence	11 (61.1%)	3 (37.5%)	8 (80.0%)			
Thrombus, %						
Presence	4 (22.2%)	2 (25.0%)	2 (20.0%)	1.33	[0.14, 12.37]	0.800
Absence	14 (77.8%)	6 (75.0%)	8 (80.0%)			
Vasa vasorum, %						
Presence	16 (88.9%)	7 (87.5%)	9 (90.0%)	0.78	[0.04, 14.75]	0.867
Absence	2 (11.1%)	1 (12.5%)	1 (10.0%)			
Macrophage accumulation, %						
Presence	11 (61.1%)	6 (75.0%)	5 (50.0%)	3.00	[0.40, 22.71]	0.288
Absence	7 (38.9%)	2 (25.0%)	5 (50.0%)			
(After balloon angioplasty)						
Lumen area, mm						
Maximum	24.2±7.3 (18)	23.0±7.8 (8)	25.2±7.1 (10)	0.96	[0.84, 1.09]	0.514
Minimum	13.4±4.1 (18)	11.2±1.9 (8)	15.1±4.7 (10)	0.71	[0.48, 1.04]	0.077
Average	18.3±5.1 (45)	15.8±3.5 (8)	20.2±5.4 (10)	0.80	[0.63, 1.03]	0.082
Stent area, mm^2^						
Maximum	36.8±8.5 (18)	35.7±8.9 (8)	37.7±8.6 (10)	0.97	[0.87, 1.09]	0.613
Minimum	25.9±6.7 (18)	24.1±5.5 (8)	27.3±7.6 (10)	0.92	[0.79, 1.08]	0.316
Average	31.5±7.0 (18)	29.4±5.4 (8)	33.3±7.9 (10)	0.91	[0.79, 1.06]	0.243
Average neointimal thickness, mm^c^	0.7±0.1 (18)	0.8±0.1 (8)	0.7±0.13 (10)	—	[—, —]	—
Average neointimal area, mm^2^	13.2±3.0 (18)	13.5±2.5 (8)	13.0±3.5 (10)	1.06	[0.77, 1.46]	0.728
Neointimal volume, mm^3^	1,526.5±1,192.2 (18)	2,032.5±1,479.5 (8)	1,121.8±756.6 (10)	1.00	[0.49, 6.38]	0.382
Percent of covered strut, %	99.2±1.7 (18)	99.7±0.6 (8)	98.8±2.3 (10)	1.77	[0.49, 6.38]	0.382
Dissection flap area, mm						
Maximum	1.0±0.8 (17)	0.8±0.4 (7)	1.1±1.0 (10)	0.61	[0.16, 2.39]	0.479
Minimum^c^	0.1±0.1 (17)	0.1±0.0 (7)	0.1±0.1 (10)	—	[—, —]	—
Average	0.4±0.3 (17)	0.4±0.2 (7)	0.5±0.3 (10)	0.17	[0.00, 11.75]	0.415

The second column displays the OFDI parameters before and after balloon angioplasty. The OFDI parameters according to restenosis 6 months after balloon angioplasty are shown in the third and fourth columns. The odds ratio as a measurement of the relationship between restenosis 6 months after balloon angioplasty and each OFDI parameter, along with its 95% confidence interval and p value, are displayed in the fifth, sixth, and seventh columns. ^a^ Mean±standard deviation (n) for continuous variables; frequency count (%) for a categorical variable. ^b^ The p-values were calculated using a simple logistic regression. ^c^ Estimate computed by logistic regression is unstable because of complete separation. OFDI: optical frequency domain imaging; OR: odds ratio; 95%CI: 95% confidence interval; Lower: lower limit; Upper: upper limit; ISR: in-stent restenosis

### Outcome

**Tables 1** and **2** present the association between clinical and imaging characteristics and the recurrent restenosis 6 months after the balloon angioplasty. Regardless of the less statistical power, recurrent restenosis at 6 months tended to be related to the multi-layered ISR pattern (OR, 6.67; 95%CI, 0.81–54.96; p=0.078), neointimal reduction (OR, 0.71; 95%CI, 0.49–1.04; p=0.082) due to angioplasty, and MLA (OR, 0.71; 95%CI, 0.48–1.04; p=0.077) after balloon angioplasty. Lesion length is not significant but numerically higher in recurrent restenosis than in non-recurrent restenosis (142.5±96.1 vs. 84.7±38.5 mm; p=0.128).

In the subgroup of patients with a complete homogeneous ISR pattern, the mean neointimal reduction at 6 months in one patient of the recurrent restenosis group was 2.1 mm^2^, whereas in six patients of the non-recurrent restenosis group, it was 8.0 mm^2^ (p=0.299).

By contrast, in the subgroup of patients with layered and multi-layered ISR patterns, the mean neointimal reduction at 6 months in seven patients of the recurrent restenosis group was 4.4 mm^2^, whereas in four patients of the non-recurrent restenosis group, it was 5.2 mm^2^ (p=0.534).

## Discussion

The major findings of the present study are as follows: (1) Lesion length was numerically higher in the recurrent restenosis group than in the non-recurrent restenosis group; (2) recurrent restenosis at 6 months tended to be related to the multi-layered ISR pattern, neointimal reduction due to balloon angioplasty, and MLA after balloon angioplasty; and (3) in the complete homogeneous pattern subgroup analysis, the mean neointimal reduction in one patient of the recurrent restenosis group at 6 months was 2.1 mm^2^, whereas that of six patients in the non-recurrent restenosis group was 8.0 mm^2^. However, these trends were not observed in the layered and multi-layered ISR patterns (recurrent restenosis, 4.4 mm^2^ vs. non-recurrent restenosis, 5.2 mm^2^).

Recent reports revealed that the lesion length was independently associated with mid-term restenosis after SFA stenting^13,14)^ in de novo lesions. As in the present study, lesion length was numerically higher in patients with recurrent restenosis than in patients without recurrent restenosis, indicating that lesion length may be a risk factor for recurrent restenosis after balloon angioplasty for ISR and de novo lesions.

Several papers reported that post-procedural MLA was associated with recurrent restenosis after balloon angioplasty for an ISR lesion in the coronary artery.^15,16)^ Indeed, in the present study, not only MLA after balloon angioplasty but also neointimal reduction might be a risk factor for recurrent restenosis at 6 months in the SFA. Especially in the patients with a homogeneous pattern in this study, neointimal reduction 6 months after balloon angioplasty of the recurrent restenosis group was numerically lower than that of the non-recurrent restenosis group. The homogeneous lesion pathologically comprises smooth muscle cells with an extracellular matrix containing collagens and proteoglycans, indicating high neointimal maturity.^17)^ We previously reported that the neointimal tissue area reduction rate was significantly smaller in the homogeneous group than in the layered and heterogeneous groups in the coronary artery.^18)^ Indeed, in the present study of the recurrent restenosis group, the mean neointimal reduction of the patients with a homogeneous ISR pattern was numerically lower than that of patients with layered and multi-layered ISR patterns. Therefore, obtaining a larger lumen area due to aggressive balloon angioplasty might be important in terms of preventing recurrent restenosis of the homogeneous ISR lesion in the femoropopliteal artery.

Contrarily, in the layered and multi-layered ISR patterns, there are no differences in the mean neointimal reduction rate between the recurrent restenosis and non-recurrent restenosis groups. This result indicates that not only obtaining a larger lumen area by aggressive balloon angioplasty but also the use of other procedural approaches, such as covered stenting or intensive pharmacological treatment, might be required for layered and multi-layered ISR lesions in the SFA.

**Figure 4:**
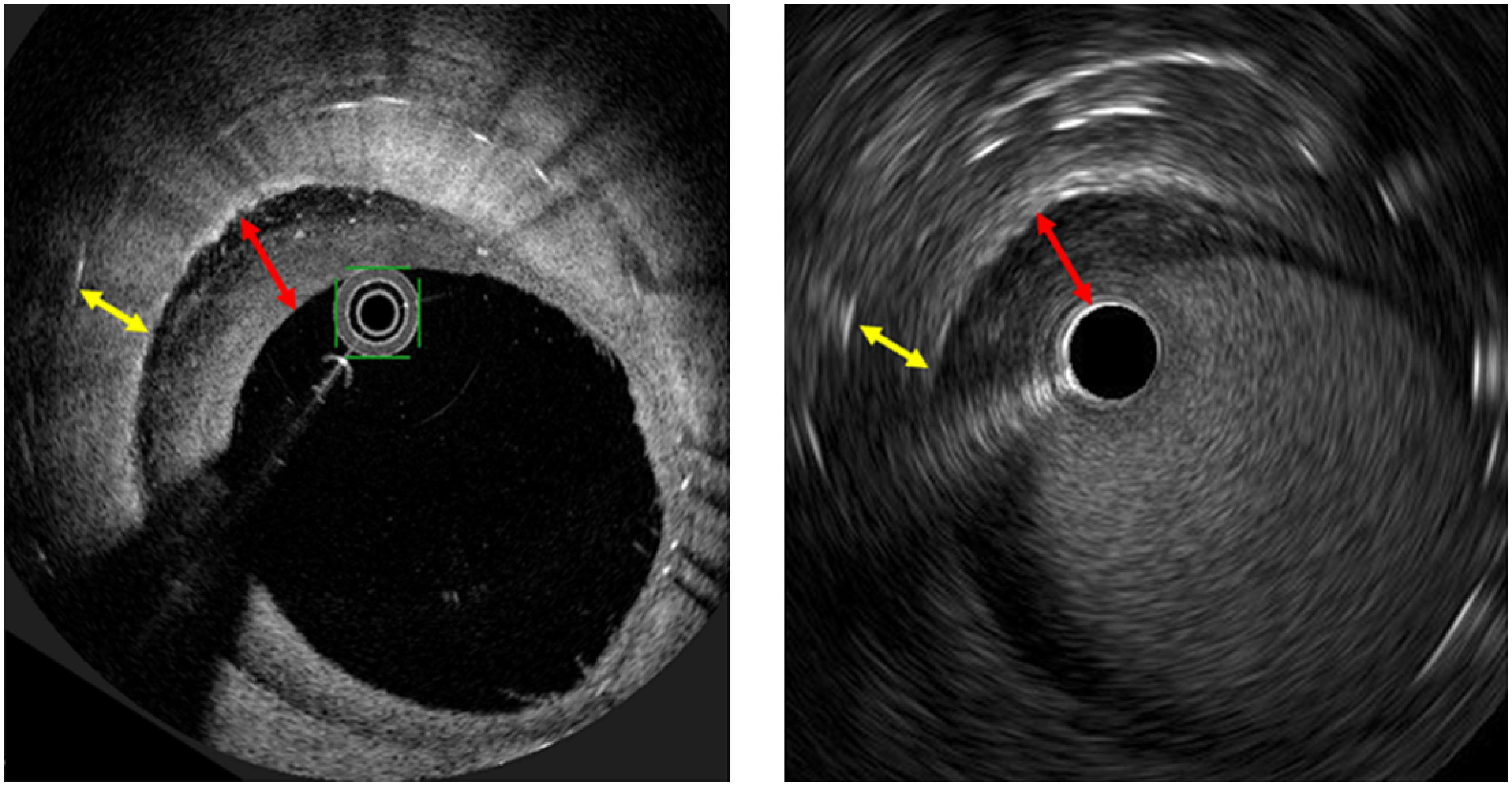
Fig. 4 Comparison of the multi-layered ISR pattern between OFDI and IVUS. (**A**) OFDI image, a superficial layer (red arrow), and a deep layer (yellow arrow). (**B**) IVUS image, a superficial hypoechoic layer (red arrow), and a deep layer (yellow arrow).

ISR: in-stent restenosis; OFDI: optical frequency domain imaging; IVUS: intravascular ultrasound

The multi-layered ISR pattern^12)^ might be a risk factor for recurrent restenosis in the present study. Clemmensen et al. reported that a multi-layered like pattern, which looks like several layers in the intima, was observed as widespread organized and layered thrombi by OCT in the native left main coronary artery.^19)^ Furthermore, Wang et al. demonstrated that the presence of a multi-layered pattern is a marker of a greater extent and severity of coronary artery disease, suggesting a pathogenic link between the plaque healing response and lesion progression.^20)^ Other studies reported that an intramural thrombus might play an important role in the development of coronary plaque with a high degree of stenosis.^21,22)^ Therefore, although mechanisms such as plaque rupture or plaque erosion have not been fully evaluated in peripheral artery disease (PAD), we speculate that thrombus formation such as a multi-layered pattern might be associated with lesion progression even in the SFA and the coronary artery. In fact, we assessed lesions with multi-layered ISR by not only OCT but also intravascular ultrasound (IVUS) in the present study, and a superficial hypoechoic layer was observed by IVUS (**Fig. 4**), suggesting widespread organized thrombus formation. A prospective study using OFDI with a larger sample size is warranted to validate our speculation.

OCT has developed into a high-resolution intracoronary imaging technology that is capable of microscopically evaluating the vessel lumen. Recent reports revealed that OCT or OFDI could provide clear images of in-stent lesions and vascular response after a drug-eluting stent in femoral-popliteal artery disease.^23)^ OFDI will be likely used to clearly visualize intravascular findings, which may have a potential in providing a better decision-making ability to improve long-term clinical outcomes. The results of a clinical trial evaluating the ability of OFDI from the SFA to the below-knee artery to expand the indications of OFDI for PAD patients receiving EVT (jRCT 2052190025)^24)^ are required.

### Limitations

One of the limitations of the present study is its small sample size, which led to the non-precise estimates of effect measures, ORs. We did not find any relationship between the examined OFDI parameters and recurrent restenosis at 6 months such that the p value <0.05. However, each parameter had a tendency of this relationship. Further studies of the ISR lesion in the SFA with sufficient sample sizes are necessary to increase our understanding of the clinical utility of OFDI.

Another limitation is that the definition of the primary endpoint (recurrent restenosis 6 months after EVT) was slightly changed before the data lock. The primary reason for the change was that the prespecified definition, solely based on PSVR >2.4, was too stringent for daily clinical practice, and some investigators did not measure the PSVR. However, the analysis of the dataset following the original definition constructed showed similar results. In this analysis, ORs [95%CI] were 8.00 [0.50 to 127.90] for the multi-layered ISR pattern, 0.56 [0.30 to 1.07] for neointimal reduction, and 0.60 [0.32 to 1.09] for MLA. Mean lesion lengths±standard deviation were 101.9±69.1 mm for recurrent restenosis and 134.1±93.0 mm for non-recurrent restenosis. In the subgroup of patients with a complete homogeneous ISR pattern, the mean neointimal reduction at 6 months in one patient of the recurrent restenosis group was 2.1 mm^2^, and in two patients of the non-recurrent restenosis group, it was 10.8 mm^2^; in the subgroup of patients with layered and multi-layered ISR patterns, the mean neointimal reduction at 6 months in seven patients of the recurrent restenosis group was 4.6 mm^2^, and in three patients of the non-recurrent restenosis group, it was 6.2 mm^2^.

Furthermore, this study only examined balloon angioplasty, warranting a study including a current medical device such as a drug-coated balloon with a larger population.

## Conclusion

The multi-layered ISR pattern and MLA after balloon angioplasty detected by OFDI might be risk factors for recurrent ISR in the SFA.
